# Social familiarity and reinforcement value: a behavioral-economic analysis of demand for social interaction with cagemate and non-cagemate female rats

**DOI:** 10.3389/fpsyg.2023.1158365

**Published:** 2023-05-12

**Authors:** Rachel Schulingkamp, Haoran Wan, Timothy D. Hackenberg

**Affiliations:** ^1^Department of Psychology, Reed College, Portland, OR, United States; ^2^Department of Psychological and Brain Sciences, Washington University in St. Louis, St. Louis, MO, United States

**Keywords:** social reinforcement, operant methods, demand analysis, rats, social familiarity

## Abstract

Rats were studied in social reinforcement procedures in which lever presses opened a door separating two adjacent spaces, permitting access to social interaction with a partner rat. The number of lever presses required for social interaction was systematically increased across blocks of sessions according to fixed-ratio schedules, generating demand functions at three different social reinforcement durations: 10 s, 30 s, and 60 s. The social partner rats were cagemates in one phase, and non-cagemates in a second phase. The rate at which social interactions were produced declined with the fixed-ratio price, and was well described by an exponential model that has been successfully employed with a range of social and non-social reinforcers. None of the main parameters of the model varied systematically with social interaction duration or with the social familiarity of the partner rat. On the whole, the results provide further evidence of the reinforcing value of social interaction, and its functional parallels with non-social reinforcers.

## Introduction

For mammals and other social animals, social interaction serves key adaptive functions at various stages of development, including parental care, social play, and reproductive behavior (Trezza et al., 2011). Indeed, this tendency to seek out social interaction is a defining characteristic of a social species. It is therefore unsurprising that social reinforcement (here defined as contingent access to social interaction) has been observed in a variety of species, including chimpanzees (Mason et al., 1962), capuchin monkeys (Dettmer and Fragaszy, 2000), horses (Søndergaard et al., 2011), foxes (Hovland et al., 2011), calves (Holm et al., 2002), sows (Kirkden and Pajor, 2006), mice (Martin et al., 2014), voles (Beery et al., 2021), hamsters (Borland et al., 2017), and rats (Wilsoncroft, 1968; Evans et al., 1994).

A variety of methods have been used to study social reinforcement (see review by Trezza et al., 2011), including operant procedures in which behavior produces opportunities for social interaction with a conspecific (Evans et al., 1994; Borland et al., 2017; Hiura et al., 2018; Vanderhooft et al., 2019; Beery et al., 2021; Hackenberg et al., 2021; Chow et al., 2022; Kirkman et al., 2022; Smith et al., 2023). Chief among the advantages of such operant methods is their analytic precision. Measuring how much work an animal will devote to obtaining social interaction, or how much it prefers one type of social interaction to another, provide quantitative assessments of relative reinforcement value. Moreover, such methods have longstanding success in the quantitative analysis of non-social reinforcers, and these can readily be brought to bear on social reinforcement effects. For example, Hiura et al. (2018) showed that social interaction with a social partner increased behavior on which it was contingent, showing a reinforcement effect; the behavior decreased when it no longer produced opportunities for social interaction, showing an extinction effect. Thus, when studied with methods with proven success in analyzing non-social reinforcement, social interaction displays characteristics of reinforcers in general.

Subsequent research with operant demand-based methods has been used to better quantify the value of social reinforcers, in much the same way such methods have been used with non-social reinforcers. In studies by Vanderhooft et al. (2019) and Chow et al. (2022), for example, rats were given repeated opportunities to produce social interaction as the fixed ratio (FR) price (number of responses to produce social interaction) increased systematically across blocks of sessions. In both studies, the frequency of social interactions varied inversely with its price, displaying the characteristic downward-sloping demand functions seen with other reinforcers (Hursh and Roma, 2016). Chow et al. (2022) also compared demand for social interaction with demand for food reinforcers, and found that while demand for both reinforcers declined with price, demand for social interaction was more sensitive to FR price changes than demand for food. Similar results were shown by Kirkman et al. (2022) using demand-based choice procedures to assess reinforcer interactions. Rats were given repeated choices between food and social interaction, with the FR prices of the two reinforcers varied, separately and together, across conditions. When the FR price of food increased while the price of social interaction was held constant, more social reinforcers were produced, suggesting that social interaction partially substituted for the higher-priced food reinforcers. Both effects were well described by an exponential model developed to quantify reinforcer value (Hursh and Silberberg, 2008), but revised to include zero levels of reinforcer production (Gilroy et al., 2021).

Prior research has also included an assessment of social reinforcement magnitude, arranged as variations in social interaction access time. Vanderhooft et al. (2019) found that in a majority of rats, the shortest (10 s) social access time produced higher levels of responding than the longest (60 s) access time. Similarly, Chow et al. (2022) found that shorter durations of social access (in the range of 15–30 s) yielded higher levels of responding, on average, than both shorter and longer durations. Complicating the interpretation of social access duration, however, are the results of a subsequent choice test, in which rats were indifferent between a shorter (3.75 s) and much longer (240 s) social access duration, suggesting that the effects of social access time may be procedure-dependent. Given such mixed results of a key reinforcement variable, additional research on social reinforcer magnitude effects is warranted.

Following Vanderhooft et al. (2019) and Chow at el. (2022), the present experiment examined demand for social interaction as a function of its duration, varied across conditions. Following Kirkman et al. (2022), we used the Gilroy et al. (2021) *Zero-Bounded Exponential* (ZBEn) model to improve the fits by handling data from sessions with zero levels of social interaction.


(1) $$IHS\left(Q_{A}\right)=IHS\left(Q_{0}\right)\times \left(e^{\frac{-\alpha }{IHS\left(Q_{0}\right)}Q_{0}P_{A}}\right)$$

where $Q_{A}$ is the consumption of commodity *A*, IHS is a log-like scale $\left(IHS=\log_{10}\left(0.5Q_{0}+\sqrt{0.25\times {Q_{0}}^{2}+1}\right)\right)$, $Q_{0}$ represents the level of demand at zero price, and *α* the rate of decline in relative consumption with increases in (FR) price, $P_{A}$, of commodity A. $P_{\text{max}}$ is defined as the price at which the slope of the demand equaled −1 (i.e., where demand changes from inelastic to elastic), and *O*_max_, the predicted consumption at P*_max_*. Together, these parameters comprise quantitative properties of reinforcer value, and are especially useful in comparisons between different reinforcer dimensions, such as reinforcer magnitude, and other variables that may influence the value of social interaction.

Another such variable is social familiarity – whether the rats are familiar with each other. Using similar operant methods, Hackenberg et al. (2021) gave rats repeated choices between interacting with a cagemate or a non-cagemate social partner. In a three-chamber apparatus, lever presses on either of two levers by a rat in the middle chamber opened an adjacent door, behind which either their cagemate or non-cagemate rat was located. Across a series of conditions and side reversals, consistent preference for the non-cagemate rat was observed (17 of 18 conditions). This type of preference for less familiar over more familiar social partners parallels findings from social preference tests in mice (Moy et al., 2004) and rats (Smith et al., 2015), although procedural differences limit more direct comparisons. In social preference tests, the social partner rats are novel each trial and thus truly unfamiliar, whereas in the operant choice procedures used by Hackenberg et al. (2021) the social partner rats serve repeatedly in that role across trials and sessions, and thus become increasingly familiar over time. Moreover, in social preference tests, animals are typically studied for only 10 min each and have only indirect contact (from behind a mesh barrier), whereas in the operant procedures used by Hackenberg et al. (2021), animals were studied for hundreds of trials over dozens of sessions, and had direct contact with one another. Notably, when social preference tests are extended to 180-min assessments, and direct contact is permitted, the novelty preference typically seen with mice and rats dissipates (Beery and Shambaugh, 2021), suggesting perhaps that brief tests with indirect contact and longer tests with direct contact are measuring different aspects of social preference. In light of these different procedures and conflicting findings, it is necessary to examine social novelty effects across different procedures.

The present study examined demand for social interaction separately for cagemate and non-cagemate rats over extended time periods – roughly 25 sessions each per social partner – and with procedures permitting direct contact between rats. While these procedures closely parallel those used by Hackenberg et al. (2021), it is not a foregone conclusion that social preferences for the non-cagemate rat will also be reflected in the present demand-based methods. In research with food and water-based reinforcers, demand-based indices of reinforcement value are not always in alignment with preference tests (Madden et al., 2007; Tan and Hackenberg, 2015). Similarly, in research on social reinforcement, Beery et al. (2021) showed that *social preference* did not always predict *social motivation*: Male prairie voles preferred familiar animals when given free access, but did not work harder to produce access to a familiar animal in an operant FR procedure, suggesting perhaps that choosing social interaction (i.e., social preference) and working to produce social interaction (i.e., social motivation) may be different aspects of socially-reinforced behavior.

Given these potential disparities across procedures, it is useful to compare behavior with social preference procedures, as in Hackenberg et al. (2021), to behavior with social motivation procedures, like those used effectively by Vanderhooft et al. (2019), Chow et al. (2022), and Kirkman et al. (2022). Based on the consistent preference for the less familiar rats reported by Hackenberg et al. (2021), one might predict that demand curves for non-cagemate rats would be less elastic (less sensitive to price) than those for more cagemate rats. If, on the other hand, the two procedures are tapping into different aspects of reinforcement value, there may be less alignment across procedures. Either way, most useful at this stage of the research is detailed parametric analyses. To that end, in the present study, we systematically explored demand and response output at three different social interaction durations (10 s, 30 s, and 60 s) for cagemate and non-cagemate rats on a within-subject basis, generating a total of 24 demand functions. This permits a quantitative behavioral-economic analysis of social reinforcement value, and how it is affected by price, social familiarity, and social reinforcement magnitude.

## Method

### Subjects

Twelve experimentally naïve female Sprague–Dawley rats (*Rattus norvegicus*) served as subjects in the present study. Rats were pair-housed in *Ancare*^®^ transparent polycarbonate rodent cages (measuring 26.5 cm × 48.2 cm × 20.3 cm) in a temperature-controlled colony room, with a 12-h light/dark cycle. Rats had free access to water, but food was restricted 18–20 h prior to each session. Four rats from different cages were arbitrarily assigned as *focal* rats (the rats with respect to which the contingencies were arranged) and the other eight rats as *social target* rats (the rats in the side chamber to which access was provided). The *social target* rats were either housed with the *focal* rats outside of the sessions (deemed *cagemate* rats) or with other rats not in the experiment (*non-cagemate* rats). All procedures were in accord with the Reed College Animal Care and Use Committee.

### Apparatus

**Figure 1:**
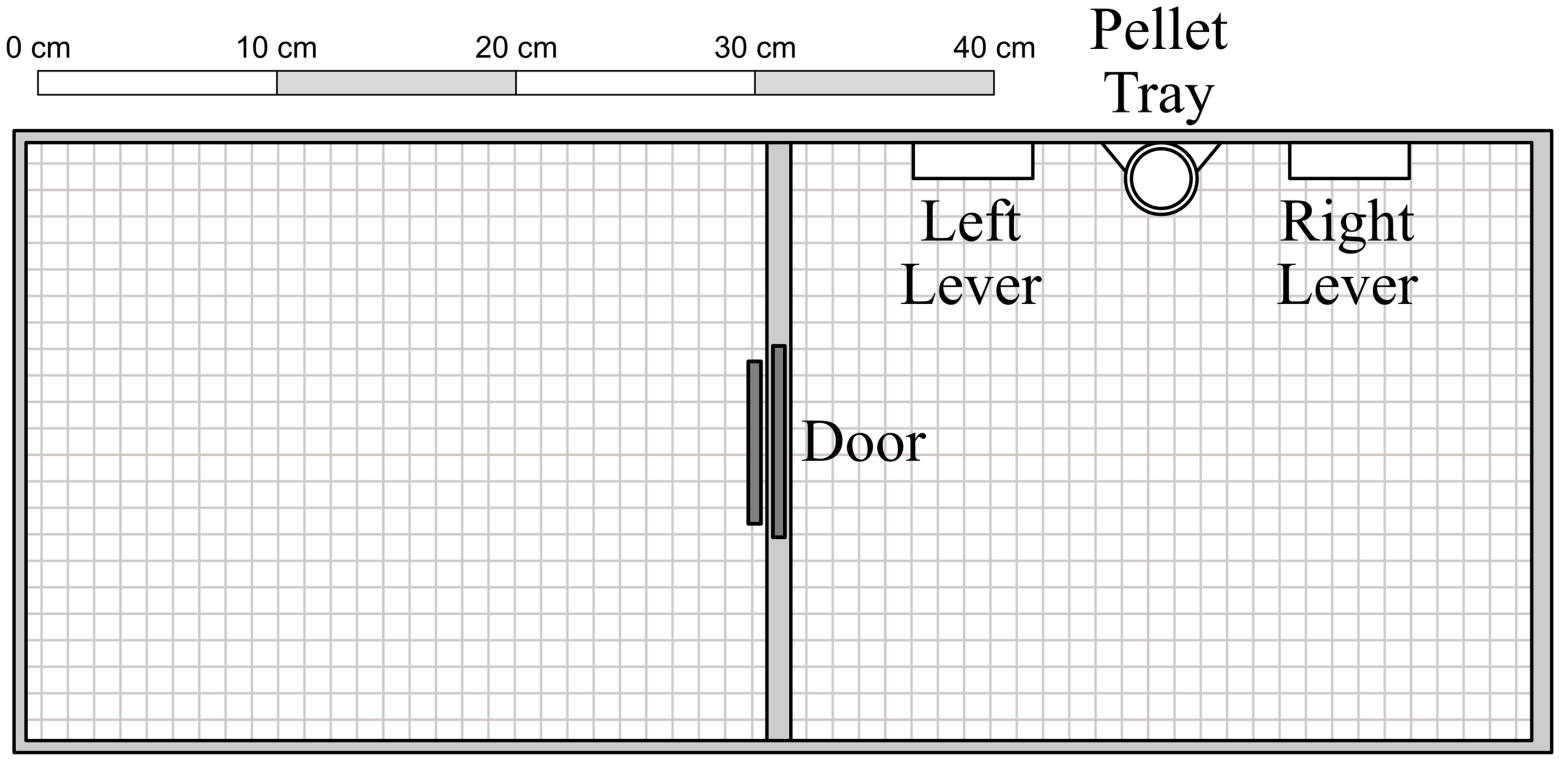
Experimental apparatus, with components presented to scale. See text for additional details.

The apparatus consisted of two adjoined chambers (31 cm × 25 cm × 22 cm) with Plexiglas barriers (see Figure 1). A circular opening (7.5 cm in diameter) was cut into the Plexiglas barriers between the left and center chambers. In its resting position, the opening was blocked by a metal door hinged at the back of the chamber; when operated, it opened upwards at a 90-degree angle. The opening was further obstructed by a flap door, designed to permit one-way access from the center to the left side chamber (but not vice versa). The center chamber contained two levers (5 cm × 1.5 cm × 1.5 cm, mounted 6 cm above the floor), and two stimulus lamps (2 cm diameter, mounted 10.5 cm above the floor), but only the left lever and stimulus lamp were used in the present experiment. Positioned 3.5 cm below and equidistant between the two levers was a food receptacle, into which 45-mg *Bio-Serv*^®^ banana-flavored sucrose pellets were delivered from a *MED Associates*^®^ pellet dispenser located behind the center wall. Experimental events were controlled and data recorded on a *Windows*^®^-based computer with *MED-PC*^®^ software. Chamber surfaces were sprayed and wiped with a sanitizer solution between sessions to reduce residual odors.

### Preliminary training

Because the rats were experimentally naïve, some preliminary training was needed prior to the experiment proper. In the initial phase (four sessions), focal rats were placed into the center chamber and the door between chambers was opened every 30 s, irrespective of responding. If the rat entered the side chamber, the door was closed. The rat was permitted 30 s to explore the side chamber, after which it was returned to the center chamber and the 30-s timer was reset. If the rat did not enter the chamber within 10 s, the door was closed and the timer reset. Initially, the flap on the door was taped open, permitting free transit to the side chamber. Once the rats were entering the side chamber consistently, we trained them to enter through the flap door by gradually lowering it across successive sessions. When the flap was at least half closed, the cagemate of the focal rat was introduced into the side chamber as a social target rat. The door continued to be opened every 30 s, and once the side chamber was entered, the rats had 30 s of social interaction time before the focal rat was returned to the center chamber. The flap continued to be lowered across successive sessions, until the focal rat entered the side chamber reliably when the flap was fully closed.

At this time, lever-press training commenced for three sessions, in which presses on the left lever (closest to the door) were reinforced by door openings, permitting social interaction with the social partner in the side chamber. This lever pressing contingency was signaled by a cue light about the left lever. The light was off during reinforcement periods, during which responses produced no scheduled consequences. This differential contingency was arranged so that the light would come to function as a discriminative stimulus (S+), signaling when lever presses would open the door, and the absence of light as an extinction stimulus (S−), signaling when lever presses were ineffective in door opening. Shaping methods were used, whereby successive approximations to lever pressing were reinforced by door opening. Such methods proved unsuccessful, however, as the social partner rats in the side chamber learned to open the flap and enter the center chamber. In an attempt to keep the rats in the side chamber, we then tried to restrain them in a harness (a method used effectively with other rats in our laboratory, Hackenberg et al., 2021), but this also failed to achieve the desired result in a timely manner, and was abandoned.

We then trained lever pressing with food rather than social access, using shaping methods described above. Establishing the food as an effective reinforcer required restricting food access outside the training sessions, and because the rats were pair housed, food was restricted for both rats for 18–20 h prior to each session. When lever pressing occurred consistently (3 sessions), the social target rat was reintroduced in the side chamber, and the consequence of lever pressing shifted from food to social interaction. Each lever press opened the door, permitting 30-s social interaction. Because the social target rats were not restrained, the social interaction period could occur in either chamber, depending on which rat initiated it. Thus, while in theory either rat could initiate the social interaction, in most cases it was initiated by the focal rat and occurred in the side chamber. When both rats were together in either chamber, the door was closed and the 30-s timer began, at the end of which the rats were returned to their respective chambers for the next trial. If neither rat initiated social interaction within 10 s, the door closed and the next trial began. Because the rats were more active and more responsive to training under food deprivation conditions, the food-restriction methods remained in place for the remainder of the experiment. This training phase was in place until responding was occurring reliably; this required nine sessions for Rats 2, 3, and 4, and 13 sessions for Rat 1.

### Experimental procedures

The main experiment involved systematic manipulations of FR price and duration of social interaction across six experimental phases. More specifically, demand functions were generated by increasing the FR price across conditions at each of three different social interaction durations (10 s, 30 s, and 60 s), first (Part 1) with cagemate rats and then (Part 2) with non-cagemate rats as social targets. Thus, FR price was manipulated across blocks of sessions (conditions), social interaction was manipulated across three different phases (blocks of conditions) and social familiarity was manipulated across two parts (blocks of phases). The demand functions in each of the six phases began with several sessions at FR 1, and thereafter increased across successive sessions until no social interactions were produced in a session. This was followed by a return to FR 1 (baseline sessions) in the subsequent phase (with a different social interaction duration). Because responding could not be maintained consistently for Rat 1 in Part 1 with the cagemate rat as social target, this rat received a single session of food reinforcement on FR 1 (with no social partner present) on the day prior to the baseline sessions in the three phases of Part 1; these food-only sessions were not needed to maintain responding in Part 2 conditions with the unfamiliar (non-cagemate) rat, and so were discontinued.

Sessions lasted 30 min, and were conducted 5 days per week at approximately the same time each day. Baseline (FR 1) conditions remained in effect until responding was deemed stable via visual inspection of daily response rates. The number of baseline sessions and FR prices could thus vary across rats, depending on how long responding took to achieve baseline stability and the FR price at which responding failed to produce social interactions. Table 1 shows the range and sequence of conditions and the number of sessions conducted at each. Due to a programming error, some of the data from the initial 30-s reinforcement duration for Rats 2, 3, and 4 were unavailable, and so this condition was replicated following the 60-s duration condition for these 3 rats.

**Table 1:** Sequence of conditions and number of experimental sessions per condition for each subject.

Rat	Phase	FR1	FR2	FR5	FR10	FR20	FR40	FR80
1	Cagemate, 10 s	4	1	1	1	–	–	–
2		8	1	1	1	1	–	–
3		3	1	1	1	1	–	–
4		6	1	1	1	1	1	–
1	Cagemate, 60 s	5	1	1	1	–	–	–
2		4	1	1	1	1	1	–
3		4	1	1	1	1	1	–
4		4	1	1	1	1	1	1
1	Cagemate, 30 s	4	1	1	1	1	–	–
2		3	1	1	1	–	–	–
3		3	1	1	1	–	–	–
4		4	1	1	1	1	1	1
1	Non-cagemate, 30 s	4	1	1	–	–	–	–
2		4	1	1	1	1	–	–
3		3	1	1	1	1	1	–
4		4	1	1	1	1	1	–
1	Non-cagemate, 10 s	7	1	1	1	1	1	–
2		3	1	1	1	1	1	–
3		6	1	1	1	1	1	1
4		4	1	1	1	1	1	1
1	Non-cagemate, 60 s	4	1	1	1	1	–	–
2		3	1	1	1	1	1	–
3		3	1	1	1	1	1	–
4		3	1	1	1	1	1	–

### Analysis

The number of social interaction episodes was recorded each session. We also recorded door openings, and while these measures could potentially differ (i.e., door openings without either rat initiating an interaction within 10 s), nearly all openings resulted in social interaction. Thus, while only social interactions (i.e., obtained reinforcers) were included in the analysis, the results would be similar had door openings (i.e., programmed reinforcers) been substituted for interactions. To account for differences in response opportunity across the different social reinforcement durations, rate of social interaction served as the main dependent variable, with active session time as the denominator. Active session time was defined as time during which a response was possible (i.e., any time the door was closed and the lever cue light on). Because active session time data were unavailable for Rats 2, 3, and 4 from the initial 30-s social reinforcement phase, only data from the replicated conditions were included in the analysis.

Social interaction rate was modeled using the ZBEn model (Eq. 1) implemented with R Version 4.2.1 (R Core Team, 2022), generating six separate curves per rat: three for social interaction duration (10, 30, and 60 s) under each of two familiarity conditions (cagemate or non-cagemate). Four parameters obtained from the demand curve model were analyzed: ***Q***_0_, the predicted consumption at zero price; ***α***, the rate of decline in relative consumption with increases in price, ***P***_*A*_; ***P***_*max*_, the predicted price at which the slope of the demand equals −1; and *O*_max_, the predicted consumption at P*_max_*. The values of ***P***_*max*_ and *O*_max_ were obtained by numeric approximation using the fitted model parameters.

## Results

**Figure 2:**
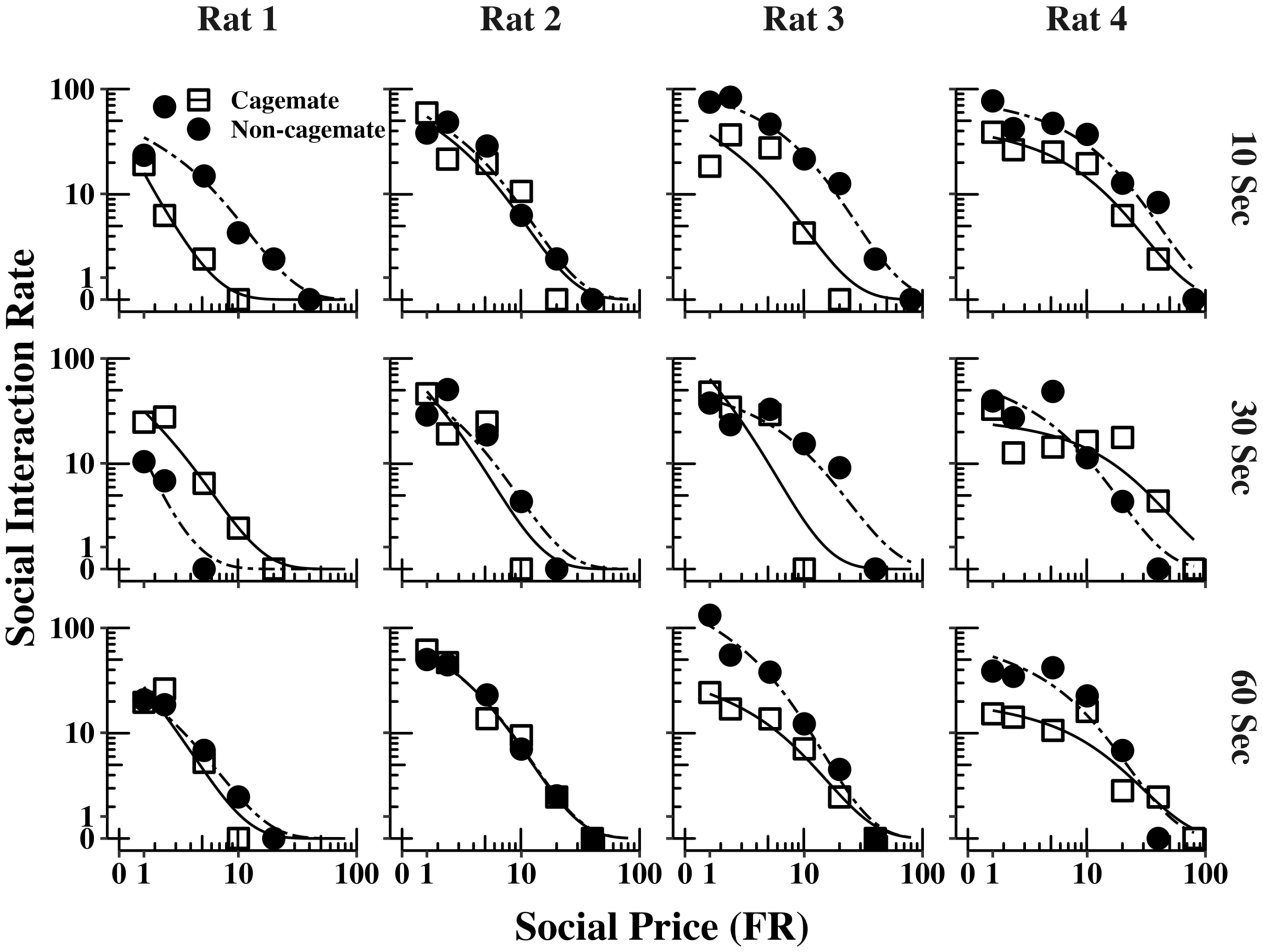
Social interaction rate (number of social interactions produced per min) as a function of FR price for each rat at all three social interaction durations, along with the fits from Eq. 1. The curves for familiar (cagemate) rats from Part 1 and for unfamiliar (non-cagemate) rats from Part 2 are indicated with different symbols.

Figure 2 shows the demand functions (social interaction rate) as a function of FR price for each rat (columns) and across the 3 social interaction durations (rows) (The response and reinforcement rates from which the demand functions were derived can be found in the Supplementary material). The demand functions obtained with cagemate and non-cagemate rats are shown as separate curves within each panel. The rate of social interaction rate declined systematically with price across all conditions for all rats, and was well described by Eq. 1, with mean *R^2^* = 0.91 (see Table 2). Data from both parts of the experiment were equally well described by Eq. 1: mean *R^2^* = 0.89 (range = 0.85–0.94) in Part 1 with cagemate rats and mean *R^2^* = 0.93 (range = 0.91–0.95) in Part 2 with non-cagemate rats.

**Table 2:** Model fits and parameter values for Eq. 1.

Rat		α		Q0		Pmax		Omax		R2	
		Cagemate	Non-cagemate	Cagemate	Non-cagemate	Cagemate	Non-cagemate	Cagemate	Non-cagemate	Cagemate	Non-cagemate
1	10 Sec	1.17E-02	2.82E-03	47.96	46.34	1.11	4.78	15.99	66.34	0.98	0.85
	30 Sec	4.72E-03	1.51E-02	55.10	53.98	2.36	1.00	39.42	12.02	0.97	0.90
	60 Sec	6.15E-03	5.90E-03	57.83	35.18	1.72	3.12	30.17	32.19	0.87	0.98
	Mean	7.52E-03	7.94E-03	53.63	45.17	1.73	2.97	28.53	36.85	0.94	0.91
2	10 Sec	2.34E-03	1.91E-03	68.03	75.03	3.77	4.15	78.81	96.17	0.87	0.97
	30 Sec	3.27E-03	2.84E-03	94.05	65.05	1.89	3.26	55.66	65.02	0.73	0.88
	60 Sec	1.86E-03	1.86E-03	82.28	79.15	3.85	4.00	98.41	98.27	0.98	0.99
	Mean	2.49E-03	2.20E-03	81.45	73.08	3.17	3.80	77.63	86.48	0.86	0.95
3	10 Sec	3.05E-03	7.61E-04	51.31	93.27	3.95	8.21	61.18	239.53	0.82	0.98
	30 Sec	2.53E-03	1.43E-03	126.63	47.53	1.77	9.20	71.31	131.11	0.75	0.87
	60 Sec	3.17E-03	1.01E-03	28.95	144.51	7.25	3.87	60.43	178.57	0.98	0.98
	Mean	2.92E-03	1.06E-03	68.96	95.10	4.32	7.09	64.31	183.07	0.85	0.94
4	10 Sec	1.31E-03	6.09E-04	39.09	73.80	12.50	13.25	144.38	301.94	0.97	0.94
	30 Sec	1.13E-03	1.58E-03	25.02	58.54	24.00	6.59	170.81	117.46	0.83	0.91
	60 Sec	2.31E-03	1.23E-03	18.01	63.73	17.07	7.69	85.28	149.99	0.89	0.90
	Mean	1.58E-03	1.14E-03	27.37	65.36	17.85	9.18	133.49	189.79	0.90	0.92

**Figure 3:**
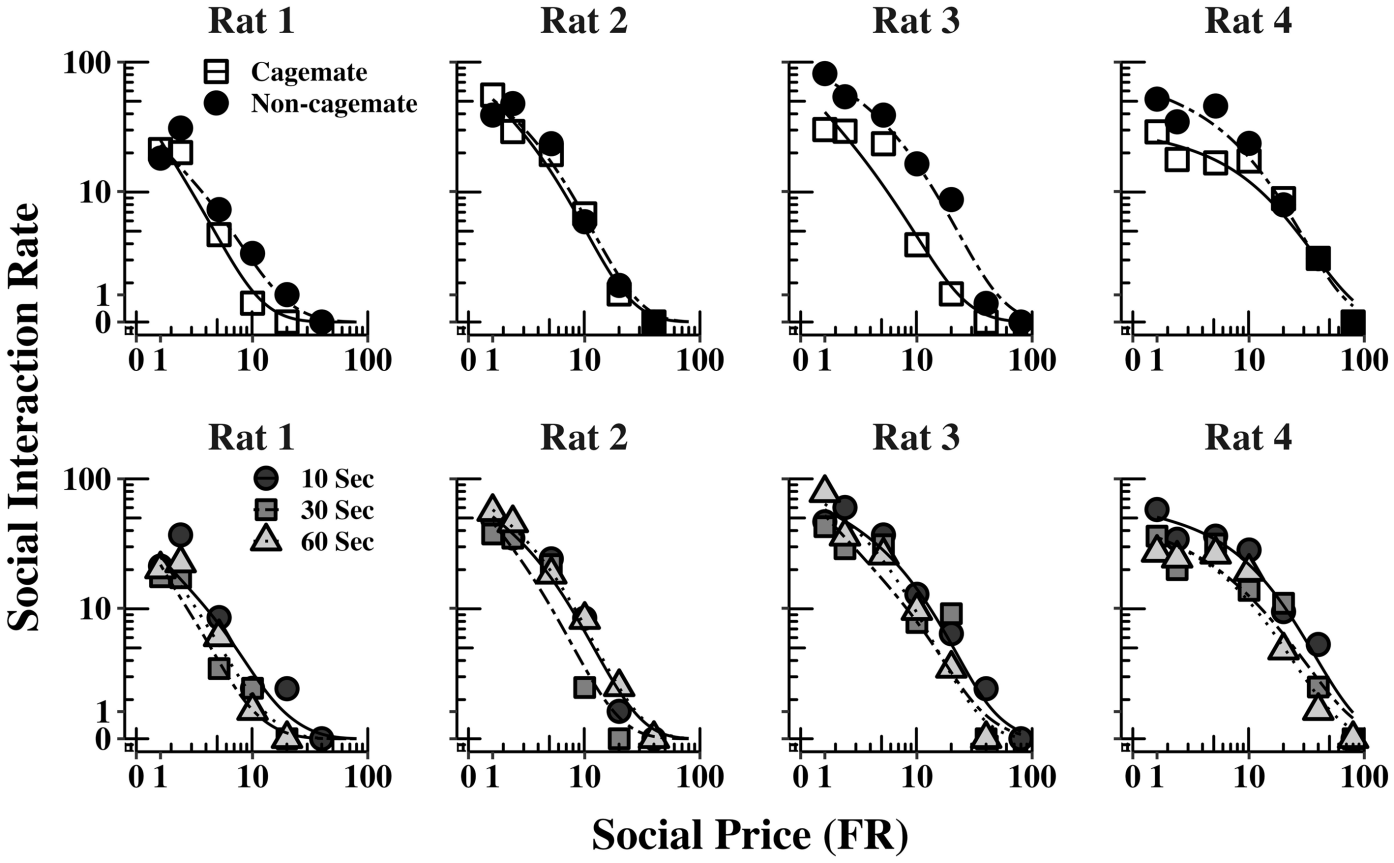
Social interaction rate (number of social interactions produced per min) as a function of FR price for each rat, collapsed across the three social interaction durations (top panels) and across social familiarity (bottom panels). The fits are from Eq. 1. See text for additional details.

Table 2 also shows parameter estimates for $Q_{0}$, *α*, $P_{\text{max}},\mathrm{and}\;O_{\text{max}}\text{,}$as defined above. None of the main parameters of the model varied systematically with either social familiarity or social interaction duration. These non-systematic effects are depicted graphically in Figure 3, which shows two sets of demand curves, one comparing Part 1 (cagemate rats) and Part 2 (non-cagemate rats), collapsed across the three social interaction durations (top panels), and one comparing the three social interaction durations, collapsed across both parts (bottom panels) (These are the same data shown in Figure 2, but aggregated differently to focus on each variable separately). Neither variable exerted consistent effects on demand for social interaction. These results were verified with linear contrast tests, averaged across the four rats. First, with respect to social familiarity (top panels), there were no significant differences in demand intensity (*Q_0_*) or demand elasticity (*α*) between Part 1, with cagemate rats, and Part 2, with non-cagemate rats, averaged across the three social interaction durations ($Q_{0}$: *p* = 0.471; *α*: *p* = 0.710). There were some exceptions for Rats 3 and 4, in that *Q_0_* was higher with non-cagemate rats (see Table 2, top panels of Figure 3), but the differences were not seen for the other two rats. Similarly, with respect to social interaction duration (see Table 2, bottom panels of Figure 3), there were also no significant differences in the main parameters of the model across the three durations, averaged across social familiarity ($Q_{0}$: *p* = 0.805; *α*: *p* = 0.226).

**Figure 4:**
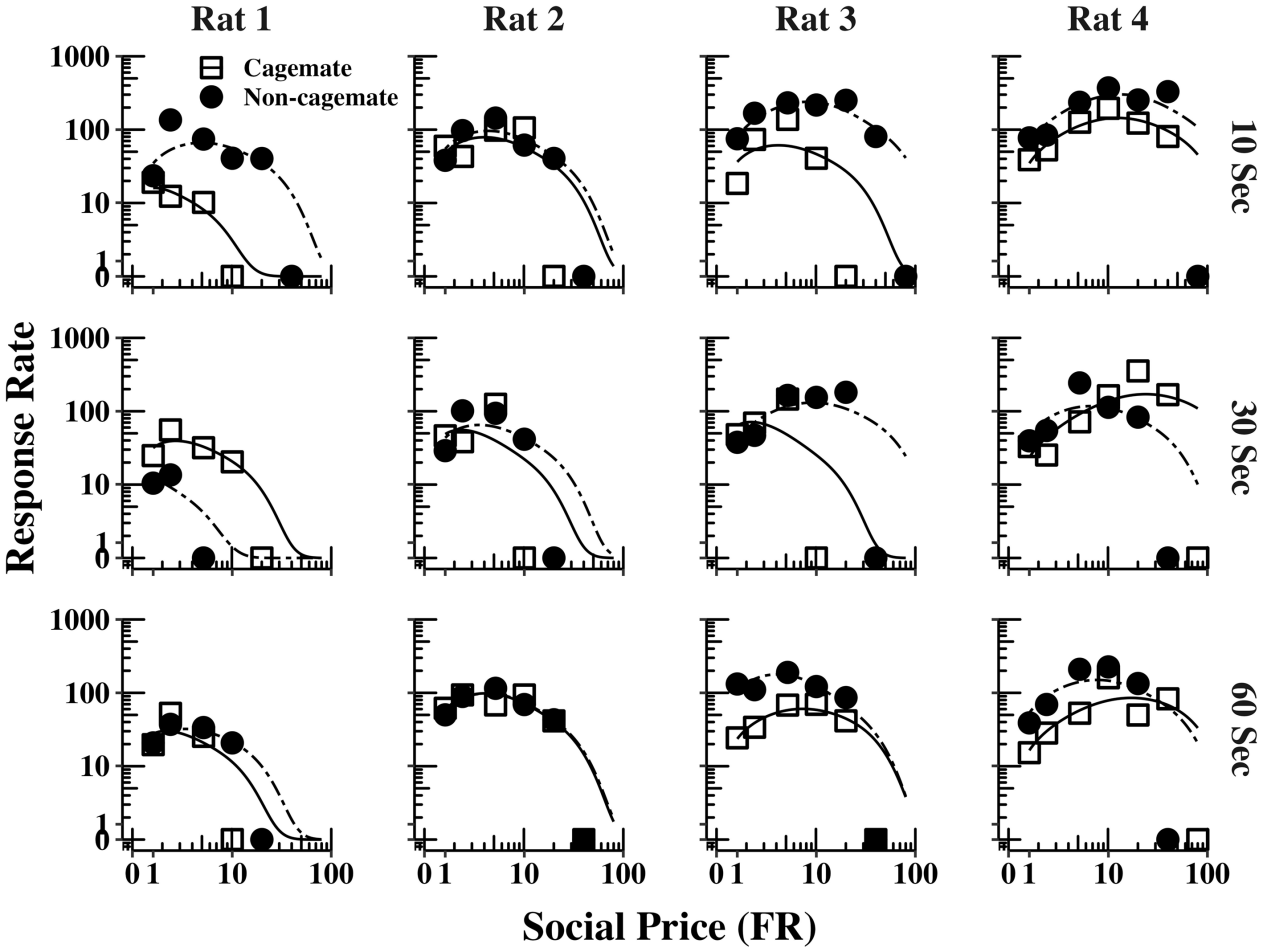
Response output (number of responses per min) as a function of FR price for each rat at all three social interaction durations, along with the fits from Eq. 1. The curves for familiar (cagemate) rats from Part 1 and for unfamiliar (non-cagemate) rats from Part 2 are indicated with different symbols.

Figure 4 shows response output (responses per min) as a function of FR price for each rat (columns) and across the three social interaction durations (rows), with curve fitting derived from Eq. 1. The functions for Part 1 (cagemates) and Part 2 (non-cagemates) are shown as separate curves within each plot. The functions were typically bitonic in form, increasing at moderate prices before declining at the higher prices, corresponding to the inelastic portions of the demand curves shown in Figure 2, before declining at the higher prices. The functions from both parts of the experiment were well described by Eq. 1, but did not vary systematically with either social familiarity or social interaction duration.

**Figure 5:**
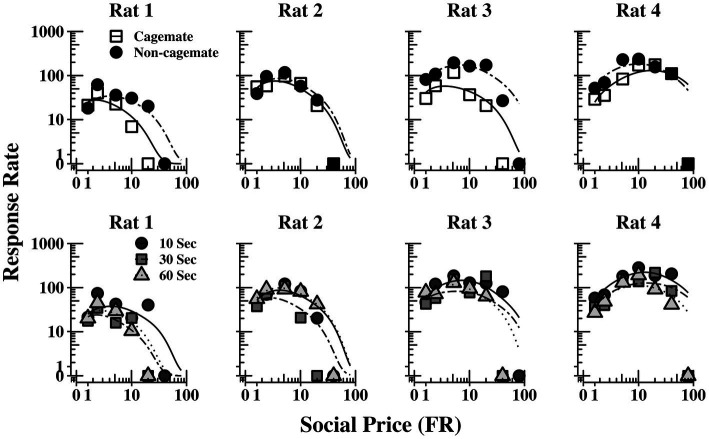
Response output (number of responses per min) as a function of FR price for each rat, collapsed across the three social interaction durations (top panels) and across social familiarity (bottom panels). The fits are from Eq. 1. See text for additional details.

Figure 5 shows the same data replotted with respect to social familiarity (top panels), collapsed across the three social interaction durations, and with respect to interaction duration (bottom panels), collapsed across social familiarity. As with the demand functions (Figure 3), the response rate functions and maximum response output (*O_max_*) were consistently higher for Rats 3 and 4 with non-cagemate rats than with cagemate rats, but these differences were not observed in the other two rats (see Table 2). Similarly, *O_max_* did not vary systematically with social interaction duration, at either the individual or group level (Figure 5 bottom, Table 2).

## Discussion

The rate at which social interaction was produced declined systematically with price, consistent with the law of demand, and with a growing body of research aimed at quantifying social reinforcement effects (Vanderhooft et al., 2019; Chow et al., 2022; Kirkman et al., 2022; Smith et al., 2023). Both the individual and aggregate data were well described by Eq. 1 (Gilroy et al., 2021), an extension of Hursh and Silberberg’s (2008) essential value model. Along with two other recent studies of social demand (Vanderhooft et al., 2019; Kirkman et al., 2022), the model accounts for over 90% of the variance describing 65 demand curves of 14 rats, across the three studies. More important than the fits *per se* is the broad consistency of the results with those of other operant-based demand methods applied to other species and other reinforcers (Hursh and Roma, 2016; Strickland and Lacy, 2020). This suggests that social reinforcement shares functional properties with other reinforcers, and demonstrates the utility of behavioral economic methods in characterizing them.

Despite the strong sensitivity to social interaction price, we did not find systematic effects of social familiarity: demand and response output for cagemate rats did not differ appreciably from demand and response output for non-cagemate rats. Some between-subject variability was evident, with hints of higher demand intensity (higher *Q_0_* values) and response output (higher *O_max_* values) for some rats responding for non-cagemates. Between-subject variability, however, was such that strong claims cannot be made. Moreover, because the demand curves for non-cagemate rats were always generated after the demand curves for cagemate rats, any effects attributable to social familiarity were confounded by order effects. Future research should include reversal conditions and between-subject counterbalancing the order of exposure to conditions, to disentangle order effects from effects of social familiarity. It would also be beneficial to replicate with a larger sample to determine whether the hints of sensitivity to social familiarity seen with some rats are reliable.

The lack of a systematic effect of social familiarity stands somewhat in contrast with prior research, in both directions – that is, both with research showing higher value of cagemate over non-cagemate (Chow et al., 2022, Experiment 2), as well as the opposite (Hackenberg et al., 2021). In the Chow et al. (2022) study, response rates were higher for cagemate than for non-cagemate rats, but only for rats housed alone outside the sessions. There were no such differences for pair-housed rats, suggesting that social motivation may have been higher for these socially isolated rats. In support of this interpretation, prior research has shown social restriction enhances social reinforcement effects (Varlinskaya and Spear, 2008; Hiura et al., 2018; Templer et al., 2018; Hackenberg et al., 2021). Such social motivation effects would presumably be less pronounced for the pair-housed rats in the present study, and may help explain the relative insensitivity to social familiarity. Future research should further explore the impact of social motivational effects on sensitivity to social familiarity.

The lack of a systematic effect of social familiarity also differs from what might be predicted on the basis of choice procedures, where consistent preference for non-cagemate over cagemate rats has been found (Hackenberg et al., 2021). Perhaps single-alternative response-output procedures used in the present study and concurrent-alternative choice procedures used in prior research tap into different aspects of social reinforcement value, as previous work with non-social reinforcers has shown (Madden et al., 2007; Tan and Hackenberg, 2015). Our initial plan was to include a concurrent-choice phase following the completion of the demand curves, permitting direct within-subject comparisons. Although time constraints precluded this part of the study, a direct comparison of demand and choice procedures within the same study should be a major priority for future research.

Especially promising in this regard would be concurrent procedures used to assess cross-price demand elasticity, which have proven useful in assessing interactions between qualitatively different reinforcers, such as social interaction and cocaine (Smith et al., 2023) and social interaction and food (Kirkman et al., 2022). In some conditions in the Kirkman et al. (2022) study, for example, the price of food reinforcement increased while the price of social interaction was held constant at FR 1. As the price of food increased, demand for food declined (own-price elasticity), while demand for social interaction increased (cross-price elasticity). That is, as food became more expensive and demand more elastic, social interaction served as a partial substitute (Green and Freed, 1993) for the more expensive food reinforcers. Such procedures would be especially useful in a quantitative assessment of own-price and cross-price elasticity with cagemate and non-cagemate rats. In the prior research showing preference for non-cagemate rats (Hackenberg et al., 2021), the costs of social access were small (i.e., essentially only the first part of a demand curve) and preferences were non-exclusive (suggesting perhaps some degree of substitution). Exploring the full demand curves with cagemate and non-cagemate rats would permit a more precise analysis of the degree to which each type of social interaction comes to substitute for the other as their price and availability changes.

In the present study, and in most prior research, social familiarity has been defined in terms of homecage housing conditions: cagemates (more familiar) or non-cagemates (less familiar). It may be more useful, however, to view social familiarity on a continuum, ranging from an unfamiliar stranger at one end to a familiar live-in partner at the other. In short-term procedures like the 10-min social preference (Smith et al., 2015), the unfamiliar rat is truly novel. In long-term procedures like the present, however, the non-cagemate rats are unfamiliar only at the beginning of the experiment; over time and experience with the procedures, they become increasingly more familiar. Our focal rats, for instance, accumulated dozens of interactions with the non-cagemate rat across Part 2 of the experiment (25 sessions, on average, per rat). Clearly the non-cagemate rats became quite familiar to the focal rats, even if somewhat less so than the cagemate rats with whom they lived outside the sessions. Perhaps the differences between a familiar cagemate and a somewhat less familiar non-cagemate in the present study were simply too small to produce an effect. Future research should further explore a wider range of points along the social familiarity continuum between the extremes of full-time cagemate and non-cagemate.

We also found no consistent effect of social interaction duration (see bottom panels of Figures 3, 5). The reasons for the lack of sensitivity to duration are unclear. One possibility is that the present procedures were simply not sufficiently sensitive to detecting effects of social reinforcement magnitude. With food reinforcers, reinforcement magnitude effects are complex and somewhat procedure dependent (Bonem and Crossman, 1988), in some cases increasing and decreasing response rates within the same study (Reed, 1991). On the other hand, using procedures similar to those used here, Vanderhooft et al. (2019) reported higher $Q_{0}$ and $P_{\text{max}}$ values for 10-s over 60-s social access durations in a majority of rats. Chow et al. (2022) similarly found relatively shorter social access times yield higher levels of responding than longer times, suggesting some sensitivity to access duration. In subsequent choice tests, however, Chow et al.’s rats were indifferent between a short (3.75 s) and much longer (240 s) social access times. That preferences were unaffected by such vast differences in social access time suggests perhaps that much of the reinforcing effect of social access occurs in the early portions of the reinforcer period, and thereafter additional time contributes little to social reinforcer value. Given the mixed patterns of results, future research should continue to explore social interaction across a wider range of durations using procedures better suited to detecting duration effects.

Another possibility is that there is more to the magnitude of social reinforcement than merely the duration of the interaction episode. In addition to the *quantity* of social interaction, more attention should be paid to the *quality* of the social interaction episode (e.g., the types of behavior it enables). A distinction has been made in prior research between *appetitive* (social motivation and approach toward the social stimulus) and *consummatory* (social contact and engagement with the social stimulus) aspects of social interaction. The present analysis focused exclusively on the former (i.e., the conditions responsible for producing the social interaction), but a detailed analysis of the latter (i.e., the behavior within the social interaction episode) would shed meaningful light on the quality of the social interaction. Both are necessary components in a comprehensive account of social reinforcement.

In behavioral-economic demand analyses, the costs and benefits are typically operationalized in terms of effort (e.g., FR price) and reinforcers consumed, respectively. Price changes also yield concomitant changes in the delay to the reinforcer, however, so it is possible to view the costs in terms of the time between reinforcers (i.e., the interreinforcement interval) in addition to the effort per reinforcer (Schwartz and Hursh, 2022). Indeed, orderly demand functions have been reported with time rather than effort as a constraint on consumption (Bauman, 1991; Tsunematsu, 2001), and there is good reason to suspect that the time between successive social reinforcers would also yield orderly social demand functions. This could be studied by future work by substituting interval for the ratio schedules used in the present study.

It is also worth noting that when the amount of a reinforcer varies across conditions, as in the present study, costs can be computed as a unit price (i.e., FR price divided by the reinforcer magnitude), yielding a composite cost metric. This type of analysis has proven especially useful in understanding drug effects on demand, where reinforcer magnitude is defined in terms of drug dose (Strickland and Lacy, 2020), but can equally well be applied to reinforcer duration. We also analyzed the present data therefore with respect to unit price (i.e., FR per social access time) in addition to the simple FR price. Because there was no magnitude effect, however, the unit price analysis did not alter the main results, and so we did not pursue this further.

In the present study, we used only female rats, as we have found in our prior work with similar procedures that female rats are somewhat more responsive than male rats to social reinforcement effects (Vanderhooft et al., 2019). Prior research with males rats, however, shows that social reinforcement effects are not limited to females (Hiura et al., 2018; Heslin and Brown, 2021; Smith et al., 2023). Indeed, Chow et al. (2022) compared male and female rats in the same experiment, and found no differences in response rate or preference measures. Similarly, strain differences do not seem to matter, as both Long Evans and Sprague–Dawley strains (the two most common rat strains studied in laboratory experiments) have been used successfully in social reinforcement research. Nonetheless, future research should continue to explore the conditions under which sex and strain differences may be seen.

Some previous research showing social reinforcement effects has used a social-release procedure, in which the social target rat is restrained in a tube and then released for a period of social interaction (Silberberg et al., 2014; Hiura et al., 2018; Vanderhooft et al., 2019; Wan et al., 2021; Kirkman et al., 2022). The use of the tube restraint grew out of earlier research claiming that social release was due not to social reinforcement, but rather, to an empathic concern for the restrained rat (Ben-Ami Bartal et al., 2011). The weight of evidence now clearly favors a social reinforcement view, not only on social-release procedures (Hachiga et al., 2018; Blystad, 2019; Heslin and Brown, 2021; Blystad and Hansen, 2022), but also on social-approach procedures like those used in the present study and by Hackenberg et al. (2021). Indeed, it is not even clear what an empathy-based account would have to say about social approach, in which the animals are unrestrained and not in any obvious state of distress that is required by an empathy-based account. A social reinforcement account, on the other hand, readily accommodates data from social-approach procedures, and indeed, from any procedures that involve contingent access to social stimuli, including mazes and place preference tasks (Trezza et al., 2011).

The social-approach methods used here also better approximate naturalistic conditions in which rats encounter one another outside the laboratory. The procedures thus have greater ecological realism than social-release procedures, in which each social access period begins with the social stimulus rat inside a tube restraint, or social-preference tests, in which the social stimulus rat is behind a mesh partition. A potential concern with the social-approach methods used here was that, because they do not specify the location or behavior of the social stimulus rat at the start of the social access period, social contact may be delayed and therefore less reinforcing than in social-release methods, in which the social interaction is initiated in the same way every trial. A related concern was that because the social stimulus rats learned to operate the flap door and thus could initiate the social contact with the focal rat, that the results may somehow differ from prior results in which the social contact was unidirectional. These concerns were unfounded, however, as the reinforcing effects of social interaction were comparable in the present study with other recent findings with social-release methods (Vanderhooft et al., 2019; Kirkman et al., 2022). The enhanced ecological realism that comes from social approach methods thus fortunately does not come at the expense of quantitative rigor.

In sum, we found that opportunities for social interaction served as effective reinforcers for rats, adding to an expanding literature on social reinforcement effects in rodents (Borland et al., 2017; Vanderhooft et al., 2019; Beery et al., 2021; Wan et al., 2021; Kirkman et al., 2022; Vahaba et al., 2022; Smith et al., 2023). Future research should include a wider range of species, including but not limited to rodents. Even amongst rodents, there are notable inter-species differences in social relationships related to differences in social ecology (Beery and Shambaugh, 2021), and a broader comparative approach that includes non-traditional species (i.e., those not typically studied in laboratory environments) is essential to a comprehensive understanding of social behavior in all its diversity (Taborsky et al., 2015). At the same time, the use of more traditional laboratory species, such as rats, can play a significant role as well, elucidating proximal mechanisms, as both the behavior and neurobiology of rats are well studied. And when combined with a functional approach regarding the evolution and social ecology of the species (Schweinfurth, 2020), the laboratory analysis of a traditional model organism like the rat can make meaningful contributions to a comparative approach. Standardized methods for analyzing and quantifying social motivation, like those used in the present study, should prove especially valuable tools in building the types of predictive testable models needed for an integrated cross-species approach to social behavior (Taborsky et al., 2015).

## Data availability statement

The original contributions presented in the study are included in the article/Supplementary material. Further inquiries can be directed to the corresponding author.

## Ethics statement

The animal study was reviewed and approved by Reed College Animal Care and Use Committee.

## Author contributions

RS and TH collaborated in the design and conduct of the experiment. RS, TH, and HW collaborated on the analyses and contributed to the writeup. All authors contributed to the article and approved the submitted version.

## Funding

The research was part of a Senior Thesis by RS, and was supported in part by a Research Initiative Grant from Reed College. The authors are indebted to Greg Wilkinson for his expert technical assistance, and to Jon Rork for comments on an earlier version of the paper. All procedures were in accord with the Reed College Institutional Animal Care and Use Committee.

## Conflict of interest

The authors declare that the research was conducted in the absence of any commercial or financial relationships that could be construed as a potential conflict of interest.

## Publisher’s note

All claims expressed in this article are solely those of the authors and do not necessarily represent those of their affiliated organizations, or those of the publisher, the editors and the reviewers. Any product that may be evaluated in this article, or claim that may be made by its manufacturer, is not guaranteed or endorsed by the publisher.
