# Supplementary material for: Social familiarity and reinforcement value: a behavioral-economic analysis of demand for social interaction with cagemate and non-cagemate female rats
Source: Front Psychol. 2023 May 12;14:1158365. doi: 10.3389/fpsyg.2023.1158365 (PMC10213623; doi:10.3389/fpsyg.2023.1158365)
Supplement: Supplementary file 1 [file Table_1.docx]

| Appendix |  |  |  |  |
| --- | --- | --- | --- | --- |
| Rat | Social Familiarity | Social Duration | Social FR | Interaction Rate |
| 1 | Cagemate | 10 Sec | 1 | 36.50 |
| 1 | Cagemate | 10 Sec | 1 | 12.56 |
| 1 | Cagemate | 10 Sec | 1 | 14.81 |
| 1 | Cagemate | 10 Sec | 1 | 12.54 |
| 1 | Cagemate | 10 Sec | 2 | 6.14 |
| 1 | Cagemate | 10 Sec | 5 | 2.02 |
| 1 | Cagemate | 10 Sec | 10 | 0.00 |
| 1 | Cagemate | 30 Sec | 1 | 18.86 |
| 1 | Cagemate | 30 Sec | 1 | 27.27 |
| 1 | Cagemate | 30 Sec | 1 | 18.75 |
| 1 | Cagemate | 30 Sec | 1 | 34.32 |
| 1 | Cagemate | 30 Sec | 2 | 27.81 |
| 1 | Cagemate | 30 Sec | 5 | 6.36 |
| 1 | Cagemate | 30 Sec | 10 | 2.04 |
| 1 | Cagemate | 30 Sec | 20 | 0.00 |
| 1 | Cagemate | 60 Sec | 1 | 36.51 |
| 1 | Cagemate | 60 Sec | 1 | 17.31 |
| 1 | Cagemate | 60 Sec | 1 | 15.66 |
| 1 | Cagemate | 60 Sec | 1 | 7.82 |
| 1 | Cagemate | 60 Sec | 1 | 20.72 |
| 1 | Cagemate | 60 Sec | 2 | 26.30 |
| 1 | Cagemate | 60 Sec | 5 | 5.10 |
| 1 | Cagemate | 60 Sec | 10 | 0.00 |
| 1 | Non-cagemate | 10 Sec | 1 | 12.72 |
| 1 | Non-cagemate | 10 Sec | 1 | 2.02 |
| 1 | Non-cagemate | 10 Sec | 1 | 12.96 |
| 1 | Non-cagemate | 10 Sec | 1 | 12.67 |
| 1 | Non-cagemate | 10 Sec | 1 | 32.47 |
| 1 | Non-cagemate | 10 Sec | 1 | 76.36 |
| 1 | Non-cagemate | 10 Sec | 1 | 15.06 |
| 1 | Non-cagemate | 10 Sec | 2 | 67.82 |
| 1 | Non-cagemate | 10 Sec | 5 | 14.87 |
| 1 | Non-cagemate | 10 Sec | 10 | 4.07 |
| 1 | Non-cagemate | 10 Sec | 20 | 2.02 |
| 1 | Non-cagemate | 10 Sec | 40 | 0.00 |
| 1 | Non-cagemate | 30 Sec | 1 | 8.86 |
| 1 | Non-cagemate | 30 Sec | 1 | 14.02 |
| 1 | Non-cagemate | 30 Sec | 1 | 7.23 |
| 1 | Non-cagemate | 30 Sec | 1 | 11.38 |
| 1 | Non-cagemate | 30 Sec | 2 | 6.74 |
| 1 | Non-cagemate | 30 Sec | 5 | 0.00 |
| 1 | Non-cagemate | 60 Sec | 1 | 6.81 |
| 1 | Non-cagemate | 60 Sec | 1 | 22.41 |
| 1 | Non-cagemate | 60 Sec | 1 | 22.43 |
| 1 | Non-cagemate | 60 Sec | 1 | 31.22 |
| 1 | Non-cagemate | 60 Sec | 2 | 18.58 |
| 1 | Non-cagemate | 60 Sec | 5 | 6.72 |
| 1 | Non-cagemate | 60 Sec | 10 | 2.07 |
| 1 | Non-cagemate | 60 Sec | 20 | 0.00 |
| 2 | Cagemate | 10 Sec | 1 | 18.46 |
| 2 | Cagemate | 10 Sec | 1 | 70.83 |
| 2 | Cagemate | 10 Sec | 1 | 58.11 |
| 2 | Cagemate | 10 Sec | 1 | 85.78 |
| 2 | Cagemate | 10 Sec | 1 | 72.07 |
| 2 | Cagemate | 10 Sec | 1 | 57.60 |
| 2 | Cagemate | 10 Sec | 1 | 69.99 |
| 2 | Cagemate | 10 Sec | 1 | 42.00 |
| 2 | Cagemate | 10 Sec | 2 | 21.54 |
| 2 | Cagemate | 10 Sec | 5 | 19.64 |
| 2 | Cagemate | 10 Sec | 10 | 10.58 |
| 2 | Cagemate | 10 Sec | 20 | 0.00 |
| 2 | Cagemate | 30 Sec | 1 | 46.68 |
| 2 | Cagemate | 30 Sec | 1 | 50.17 |
| 2 | Cagemate | 30 Sec | 1 | 41.89 |
| 2 | Cagemate | 30 Sec | 2 | 19.17 |
| 2 | Cagemate | 30 Sec | 5 | 24.90 |
| 2 | Cagemate | 30 Sec | 10 | 0.00 |
| 2 | Cagemate | 60 Sec | 1 | 58.33 |
| 2 | Cagemate | 60 Sec | 1 | 63.60 |
| 2 | Cagemate | 60 Sec | 1 | 73.58 |
| 2 | Cagemate | 60 Sec | 1 | 50.92 |
| 2 | Cagemate | 60 Sec | 2 | 46.85 |
| 2 | Cagemate | 60 Sec | 5 | 13.67 |
| 2 | Cagemate | 60 Sec | 10 | 9.35 |
| 2 | Cagemate | 60 Sec | 20 | 2.07 |
| 2 | Cagemate | 60 Sec | 40 | 0.00 |
| 2 | Non-cagemate | 10 Sec | 1 | 36.25 |
| 2 | Non-cagemate | 10 Sec | 1 | 36.18 |
| 2 | Non-cagemate | 10 Sec | 1 | 42.12 |
| 2 | Non-cagemate | 10 Sec | 2 | 48.47 |
| 2 | Non-cagemate | 10 Sec | 5 | 28.74 |
| 2 | Non-cagemate | 10 Sec | 10 | 6.15 |
| 2 | Non-cagemate | 10 Sec | 20 | 2.02 |
| 2 | Non-cagemate | 10 Sec | 40 | 0.00 |
| 2 | Non-cagemate | 30 Sec | 1 | 34.16 |
| 2 | Non-cagemate | 30 Sec | 1 | 6.35 |
| 2 | Non-cagemate | 30 Sec | 1 | 54.87 |
| 2 | Non-cagemate | 30 Sec | 1 | 20.59 |
| 2 | Non-cagemate | 30 Sec | 2 | 50.66 |
| 2 | Non-cagemate | 30 Sec | 5 | 18.75 |
| 2 | Non-cagemate | 30 Sec | 10 | 4.15 |
| 2 | Non-cagemate | 30 Sec | 20 | 0.00 |
| 2 | Non-cagemate | 60 Sec | 1 | 63.85 |
| 2 | Non-cagemate | 60 Sec | 1 | 42.08 |
| 2 | Non-cagemate | 60 Sec | 1 | 44.34 |
| 2 | Non-cagemate | 60 Sec | 2 | 44.65 |
| 2 | Non-cagemate | 60 Sec | 5 | 23.06 |
| 2 | Non-cagemate | 60 Sec | 10 | 6.92 |
| 2 | Non-cagemate | 60 Sec | 20 | 2.15 |
| 2 | Non-cagemate | 60 Sec | 40 | 0.00 |
| 3 | Cagemate | 10 Sec | 1 | 21.57 |
| 3 | Cagemate | 10 Sec | 1 | 12.71 |
| 3 | Cagemate | 10 Sec | 1 | 21.00 |
| 3 | Cagemate | 10 Sec | 2 | 36.71 |
| 3 | Cagemate | 10 Sec | 5 | 27.59 |
| 3 | Cagemate | 10 Sec | 10 | 4.07 |
| 3 | Cagemate | 10 Sec | 20 | 0.00 |
| 3 | Cagemate | 30 Sec | 1 | 49.91 |
| 3 | Cagemate | 30 Sec | 1 | 33.42 |
| 3 | Cagemate | 30 Sec | 1 | 60.48 |
| 3 | Cagemate | 30 Sec | 2 | 34.46 |
| 3 | Cagemate | 30 Sec | 5 | 29.29 |
| 3 | Cagemate | 30 Sec | 10 | 0.00 |
| 3 | Cagemate | 60 Sec | 1 | 42.48 |
| 3 | Cagemate | 60 Sec | 1 | 15.41 |
| 3 | Cagemate | 60 Sec | 1 | 19.27 |
| 3 | Cagemate | 60 Sec | 1 | 20.02 |
| 3 | Cagemate | 60 Sec | 2 | 16.94 |
| 3 | Cagemate | 60 Sec | 5 | 13.61 |
| 3 | Cagemate | 60 Sec | 10 | 6.96 |
| 3 | Cagemate | 60 Sec | 20 | 2.08 |
| 3 | Cagemate | 60 Sec | 40 | 0.00 |
| 3 | Non-cagemate | 10 Sec | 1 | 14.86 |
| 3 | Non-cagemate | 10 Sec | 1 | 33.75 |
| 3 | Non-cagemate | 10 Sec | 1 | 68.30 |
| 3 | Non-cagemate | 10 Sec | 1 | 126.11 |
| 3 | Non-cagemate | 10 Sec | 1 | 119.48 |
| 3 | Non-cagemate | 10 Sec | 1 | 88.05 |
| 3 | Non-cagemate | 10 Sec | 2 | 83.66 |
| 3 | Non-cagemate | 10 Sec | 5 | 46.37 |
| 3 | Non-cagemate | 10 Sec | 10 | 21.65 |
| 3 | Non-cagemate | 10 Sec | 20 | 12.55 |
| 3 | Non-cagemate | 10 Sec | 40 | 2.01 |
| 3 | Non-cagemate | 10 Sec | 80 | 0.00 |
| 3 | Non-cagemate | 30 Sec | 1 | 44.48 |
| 3 | Non-cagemate | 30 Sec | 1 | 33.50 |
| 3 | Non-cagemate | 30 Sec | 1 | 35.12 |
| 3 | Non-cagemate | 30 Sec | 2 | 23.46 |
| 3 | Non-cagemate | 30 Sec | 5 | 32.86 |
| 3 | Non-cagemate | 30 Sec | 10 | 15.45 |
| 3 | Non-cagemate | 30 Sec | 20 | 9.06 |
| 3 | Non-cagemate | 30 Sec | 40 | 0.00 |
| 3 | Non-cagemate | 60 Sec | 1 | 114.89 |
| 3 | Non-cagemate | 60 Sec | 1 | 133.86 |
| 3 | Non-cagemate | 60 Sec | 1 | 147.10 |
| 3 | Non-cagemate | 60 Sec | 2 | 55.20 |
| 3 | Non-cagemate | 60 Sec | 5 | 37.97 |
| 3 | Non-cagemate | 60 Sec | 10 | 12.18 |
| 3 | Non-cagemate | 60 Sec | 20 | 4.30 |
| 3 | Non-cagemate | 60 Sec | 40 | 0.00 |
| 4 | Cagemate | 10 Sec | 1 | 6.14 |
| 4 | Cagemate | 10 Sec | 1 | 47.01 |
| 4 | Cagemate | 10 Sec | 1 | 56.96 |
| 4 | Cagemate | 10 Sec | 1 | 34.15 |
| 4 | Cagemate | 10 Sec | 1 | 57.40 |
| 4 | Cagemate | 10 Sec | 1 | 31.68 |
| 4 | Cagemate | 10 Sec | 2 | 26.45 |
| 4 | Cagemate | 10 Sec | 5 | 25.22 |
| 4 | Cagemate | 10 Sec | 10 | 19.53 |
| 4 | Cagemate | 10 Sec | 20 | 6.14 |
| 4 | Cagemate | 10 Sec | 40 | 2.02 |
| 4 | Cagemate | 30 Sec | 1 | 33.68 |
| 4 | Cagemate | 30 Sec | 1 | 44.20 |
| 4 | Cagemate | 30 Sec | 1 | 12.78 |
| 4 | Cagemate | 30 Sec | 1 | 41.06 |
| 4 | Cagemate | 30 Sec | 2 | 12.62 |
| 4 | Cagemate | 30 Sec | 5 | 14.34 |
| 4 | Cagemate | 30 Sec | 10 | 16.28 |
| 4 | Cagemate | 30 Sec | 20 | 17.72 |
| 4 | Cagemate | 30 Sec | 40 | 4.20 |
| 4 | Cagemate | 30 Sec | 80 | 0.00 |
| 4 | Cagemate | 60 Sec | 1 | 21.82 |
| 4 | Cagemate | 60 Sec | 1 | 6.71 |
| 4 | Cagemate | 60 Sec | 1 | 12.14 |
| 4 | Cagemate | 60 Sec | 1 | 20.19 |
| 4 | Cagemate | 60 Sec | 2 | 14.13 |
| 4 | Cagemate | 60 Sec | 5 | 10.54 |
| 4 | Cagemate | 60 Sec | 10 | 16.04 |
| 4 | Cagemate | 60 Sec | 20 | 2.49 |
| 4 | Cagemate | 60 Sec | 40 | 2.07 |
| 4 | Cagemate | 60 Sec | 80 | 0.00 |
| 4 | Non-cagemate | 10 Sec | 1 | 53.27 |
| 4 | Non-cagemate | 10 Sec | 1 | 102.50 |
| 4 | Non-cagemate | 10 Sec | 1 | 95.44 |
| 4 | Non-cagemate | 10 Sec | 1 | 57.67 |
| 4 | Non-cagemate | 10 Sec | 2 | 42.08 |
| 4 | Non-cagemate | 10 Sec | 5 | 47.21 |
| 4 | Non-cagemate | 10 Sec | 10 | 37.08 |
| 4 | Non-cagemate | 10 Sec | 20 | 12.68 |
| 4 | Non-cagemate | 10 Sec | 40 | 8.24 |
| 4 | Non-cagemate | 10 Sec | 80 | 0.00 |
| 4 | Non-cagemate | 30 Sec | 1 | 60.73 |
| 4 | Non-cagemate | 30 Sec | 1 | 39.07 |
| 4 | Non-cagemate | 30 Sec | 1 | 32.09 |
| 4 | Non-cagemate | 30 Sec | 1 | 26.09 |
| 4 | Non-cagemate | 30 Sec | 2 | 27.32 |
| 4 | Non-cagemate | 30 Sec | 5 | 48.65 |
| 4 | Non-cagemate | 30 Sec | 10 | 11.31 |
| 4 | Non-cagemate | 30 Sec | 20 | 4.15 |
| 4 | Non-cagemate | 30 Sec | 40 | 0.00 |
| 4 | Non-cagemate | 60 Sec | 1 | 39.05 |
| 4 | Non-cagemate | 60 Sec | 1 | 37.25 |
| 4 | Non-cagemate | 60 Sec | 1 | 40.45 |
| 4 | Non-cagemate | 60 Sec | 2 | 34.68 |
| 4 | Non-cagemate | 60 Sec | 5 | 41.86 |
| 4 | Non-cagemate | 60 Sec | 10 | 22.57 |
| 4 | Non-cagemate | 60 Sec | 20 | 6.70 |
| 4 | Non-cagemate | 60 Sec | 40 | 0.00 |
